# The representation of bullying in Italian primary school children: A mixed-method study comparing drawing and interview data and their association with self-report involvement in bullying events

**DOI:** 10.3389/fpsyg.2022.862711

**Published:** 2022-09-06

**Authors:** Davide Marengo, Michele Settanni, Claudio Longobardi, Matteo Angelo Fabris

**Affiliations:** Department of Psychology, University of Turin, Turin, Italy

**Keywords:** bullying, victims, children’s drawing, interview, primary school

## Abstract

Bullying continues to be a social issue affecting millions of students of all ages worldwide. Research on bullying seems to be dominated by quantitative research approaches employed standardized categories and measures, ultimately limiting our knowledge about children’s own view on bullying. Our research follows another direction, aiming to explore the representation of bullying in a sample of Italian primary school children by using and comparing the functioning of two qualitative research instruments: interviews, and children’s drawings. In addition, aided by quantitative analyses, we aimed to investigate whether students’ involvement in different bullying roles (as bullies, victims, or defenders), as measured by self-assessment, correlated with different characteristics of the representation of bullying emerging from children’s drawings and interviews. We recruited a convenient sample of 640 primary school students (mean age = 9.44; SD = 0.67), 53.3% of whom were male. The results showed that all forms of bullying, i.e., physical, verbal, and social bullying, could be identified in interview and drawing data, although references to all types of bullying were more frequent in interview data. In terms of bullying criteria, the presence of a power imbalance between the bully and the victim was most frequently detected in both the interview data and the drawing data, while repetition was more easily detected in the interview data. The interview data showed that sadness was the most frequently reported victim emotions, followed by fear, anger, and lack of emotion. The drawing data showed a similar pattern, although victims were more frequently described as lacking emotions compared to the interview data. In both interview and drawing data, age and female gender were positively associated with references to verbal bullying, and negatively associated with references to physical bullying. Additionally, bully/victim children were more likely than uninvolved children to depict physical bullying in the drawings, while this association was not detected in interview data. In summary, our study shows that, compared with drawings, interviews tend to provide a more comprehensive view of children’s own representation of bullying, while drawing data tend to show stronger connections with children’s current personal experiences of bullying.

## Introduction

School is a context in which various forms of victimization can occur (Longobardi et al., 2017, 2019; Badenes-Ribera et al., 2022). Bullying continues to be a social issue affecting millions of students of all ages worldwide (Craig et al., 2009; Ossa et al., 2021) which tends to be associated with poorer developmental and academic outcomes for affected children (Moore et al., 2017; Prino et al., 2019; Fabris et al., 2021). Bullying is usually defined as a frequent, repeated, and intentional form of aggression involving an imbalance of power or strength between the bully and the victim (Olweus and Limber, 2010) which may be exerted in different forms, including physical aggression (e.g., being hit, kicked, pushed, shoved), verbal aggression (e.g. being insulted or called nasty and hurtful names or threatened) or social exclusion (e.g., being ignored or excluded from peer groups). Bullying is also considered a group phenomenon in which one or more individuals (bullies) repeatedly and intentionally attack, humiliate, or exclude others (victims) who have difficulty fighting back (Salmivalli, 2010). The social scene of bullying is complex, and peers may participate not only as victims, bullies, or bullies-victims, but also play other roles. There are assistants to the bully who join the ringleaders to attack a victim; reinforcers who are not actively involved in the bullying but are instrumental to the actions of the bully; defenders who actively intervene and try to stop the bullying (e.g., demand teacher intervention or try to comfort the victim); and bystanders who know what is happening but do not take sides with either the bully or the victim (Salmivalli, 2010).

Although peer aggression usually peaks in early adolescence (Perry et al., 1988), forms of bullying can also occur in primary school, and some studies have found certain gender differences in the prevalence of involvement. In particular, males appear to be at heightened risk of being involved in bullying, both as bullies and as victims, while females tend to report more often forms of indirect victimization (Iossi Silva et al., 2013; Ang et al., 2018; Twardowska-Staszek et al., 2018; Jiménez, 2019) and are more likely to engage in forms of indirect bullying such as active social exclusion (Ang et al., 2018).

The majority of research currently conducted on the topic of children’s involvement in bullying appears to favor quantitative studies (Patton et al., 2017; Marengo et al., 2021; Samara et al., 2021). Quantitative studies based on self-report questionnaires allow us to increase our knowledge in large samples in a comparable way. However, they have the limitation of not revealing the subjective experiences of the children involved. Unlike quantitative surveys, qualitative research is typically inductive and therefore lends itself to an in-depth exploration of the perspectives, perceptions, and experiences of children involved in bullying (Bosacki et al., 2006; Patton et al., 2017). This is a central aspect of bullying research because it allows researchers to examine more closely how children perceive and define bullying, which has concrete implications for intervention and prevention strategies. Along these lines, qualitative research allows us to examine children’s representations of bullying, integrate and extend data from quantitative research, and thus inform researchers and practitioners about intervention and prevention strategies (Torrance, 2000; Espelage and Asidao, 2001; Patton et al., 2017). In practice, as several authors point out (Bosacki et al., 2006), quantitative research forces the child to answer questions designed and suggested by researchers, whereas qualitative research allows children to express their own perspectives and highlight the aspects of the phenomenon that are most important to them. In doing this, we may be more able recognize that there is no single, common definition of bullying and that the definition of bullying, and thus the perception of the phenomenon, may vary between students and adults, such as researchers, school staff, and teachers (Eriksen, 2018). Still, use of qualitative research is not without limitations, as it is typically more time consuming and requires more resources than quantitative research (e.g., personnel performing interviews, or the coding of collected data; Carter and Henderson, 2005). Lack of anonymity may also be another issue possibly introducing bias in the assessment procedure in terms of both lowering proneness to respond, as well as the need to do it in socially desirable way (Bergen and Labonté, 2020).

A common data collection approach in qualitative research is the use of in-person interviews (Silverman, 2016). Using interviews with primary school children, Guerin and Hennessy (2002) found that verbal and physical bullying were the most common forms of bullying in children’s narratives, followed by forms of bullying characterized by threats, spreading rumors, and social exclusion. However, in contrast to the definitions proposed by researchers, it appears that repetition, intent, and lack of provocation are not central to definitions of bullying by students (Madsen, 1996; Guerin and Hennessy, 2002; Monks and Smith, 2006; Naylor et al., 2006), while harm inflicted on victims is a salient feature in their definition of bullying (Madsen, 1996; Naylor et al., 2006).

Studies using interviews or open-ended questions also report age and gender differences in children’s representation of bullying. As for age, young children tend to differentiate only between non-aggressive and aggressive acts, viewing the latter as bullying even when they do not involve bullying behavior (e.g., children of equal power fighting over a misunderstanding); in turn adolescents and adults have a more complex understanding of bullying, successfully distinguishing between direct and indirect forms of physical, relational/social, and verbal bullying behaviors (Smith et al., 2002; Monks and Smith, 2006; Naylor et al., 2006). For example, in the school context, children are more likely to refer to direct victimization (physical and verbal) compared to their teachers, who in turn tend to refer also to indirect forms of bullying (e.g., social exclusion; Naylor et al., 2006).

When compared with males, females’ representations of bullying appear to focus more on the impact of bullying and the emotional well-being of the victims; in turn, males are more likely to describe observable behaviors that may occur in bullying incidents (Naylor et al., 2006; Byrne et al., 2016). Some data show that females tend to report more verbal abuse than males (Naylor et al., 2006). In addition, Naylor et al. (2006) report that females tend to report social exclusion as a form of bullying more often than males; however, some studies do not support this finding (Guerin and Hennessy, 2002; Smith et al., 2002).

Interviews are not the only qualitative techniques used in studying children’s representation of bullying among primary school students. Some surveys have used children’s drawings to identify children’s representations of bullying (Bosacki et al., 2006; Patton et al., 2017). Children’s drawing appears to be a useful tool for research because it allows us to examine the representations and perceptions that children exhibit toward a particular topic of inquiry (Bozzato et al., 2021). Drawing is considered an attractive and entertaining activity for children (Kukkonen and Chang-Kredl, 2018). From a methodological perspective, children’s drawing could be an investigative tool that benefits children who have difficulty with verbal expression, and through drawing, the child can incorporate elements that are important to him or her in terms of representing the phenomena he or she depicts (DiCarlo et al., 2000; MacPhail and Kinchin, 2004). In addition, several studies point to the importance of children’s drawings as a tool for assessing psychological well-being and the quality of interpersonal relationships (Bombi et al., 2007; Laghi et al., 2013; Potchebutzky et al., 2020; Kallitsoglou et al., 2022).

Research on bullying conducted using the drawing technique shows some interesting findings, which we will summarize here. The vast majority of primary school children tend to draw the victim-perpetrator dyad, while children only begin to draw more than two people in the scene as they get older (Bosacki et al., 2006). It appears that children tend to draw the bullying scene protagonists with their own gender, while only a smaller percentage (10%) draw mixed-gender scenarios (in which a male typically bullies a female) (Bosacki et al., 2006). Children usually draw bullies either larger or the same size as the victim, while it is rare for the victim to be drawn larger than the bully (Bosacki et al., 2006; Slee and Skrzypiec, 2015). Most children draw facial expressions for both the bully and the victim (Bosacki et al., 2006). For the bullies, the majority draw positive facial expressions, while only between 6 and 38% of them draw negative facial expressions (Bosacki et al., 2006; Slee and Skrzypiec, 2015). Regarding victims, the majority of them are presented with a negative facial expression and to a lesser extent with a neutral or positive facial expression (Bosacki et al., 2006; Slee and Skrzypiec, 2015). Many children also draw “speech bubbles” or verbal comments, and this appears to characterize younger children in particular (Bosacki et al., 2006). However, as Bosacki et al. (2006) note, not only do the depictions of verbal comments decrease as children get older, but older children are more likely to portray the victim as silent during bullying events. According to Andreou and Bonoti’s (2010) survey, nearly half of the children draw themselves in the bullying scene, as victim, bully, or defender, but not as helper or reinforcer of the bully. Girls tend to draw themselves into more verbal victimization scenes than boys, while boys tend to draw themselves as engaging physical acts of bullying. Furthermore, Andreou and Bonoti’s (2010) analysis shows that physical, verbal, or mixed (both physical and verbal) forms of victimization appear in the drawings, while other forms of violence, such as attacks on property or social exclusion, are not depicted.

One aspect that we believe is insufficiently explored in the literature is whether experiences with bullying (as victim, aggressor, or bystander) may be associated with children’s representation of bullying in some way. In this direction, evidence suggests that the experience of peer victimization does not appear to be associated with children’s definition of bullying in interviews (Monks and Smith, 2006; Naylor et al., 2006). However, when confronted with vignettes depicting bullying, bullies tend not to recognize these aggressive behaviors as bullying (Monks and Smith, 2006). This could be because bullies’ moral restraint leads them to minimize negative emotions (such as shame and guilt) and emphasize positive reactions to bullying (Ortega et al., 2002). This would result in aggressive acts being less recognized and defined by bullies as bullying behavior (Monks and Smith, 2006). In a large sample of adolescents, Byrne et al. (2016) found that students who had experienced peer victimization were more likely to discuss the emotional impact of bullying on the victim in their definition of bullying compared to those who had not been victimized.

Regarding the relationship between self-reported bullying involvement and drawing in childhood, we are aware of only two studies that have attempted to examine possible correlates in primary school children (Andreou and Bonoti, 2010; Slee and Skrzypiec, 2015). Andreou and Bonoti (2010) examined the correlation between self-report and bullying design and found a weak correlation. Slee and Skrzypiec (2015) found that children who were bullied tended to make more detailed drawings and depict less spatial distance between the figures of the victim and the bully. No significant relationship was found between the frequency of victimization and the size of the bully or victim and some graphic indices traditionally associated with emotional well-being, such as the size of the drawing and the weight of the lines. Overall, then, there is a need to explore the relationship between self-report and qualitative research instruments (particularly interviews and children’s drawings) to understand primary school children’s representation bullying. Furthermore, there is limited evidence of comparisons between different qualitative approaches such as drawing and interviewing to understand whether both instruments can be considered informative and whether they converge or diverge in terms of the information they provide about the child’s experience.

### The present study

Research on bullying seems to be dominated by quantitative research approaches, thus disregarding important information about children’s representation of bullying and their involvement in the phenomenon. The present study seeks to fill this gap using a mixed method approach. Using two qualitative research approaches, namely the interview and children’s drawings, we collected data about the representation of bullying (and the characteristics that define it) in a sample of Italian primary school children. Based on collected data, and aided by quantitative methods, we sought to answer multiple research questions. First, we sought to determine if children’s representations of bullying emerging from interview and drawing data differed in significant way. We based this comparison on a set of bullying characteristics naturally emerging from the data, including the type of bullying behaviors enacted by the bully (e.g., physical, verbal, and relational bullying), their compatibility with commonly used criteria for bullying (i.e., repetition, power imbalance, and intentionality), the emotional and behavioral response of the victims, and the presence of other individuals (e.g., teachers and other children). Secondly, we wanted to understand if the children’s representations of bullying observed in their drawing and interview would be related to the demographic characteristics of the children, as well as their direct involvement in bullying episodes as measured using a self-report questionnaire. Thus, asking the children to describe their personal representation of bullying through their drawing and interview data, and self-report about their involvement bullying episodes, allowed us to examine how these experiences were associated with their view on bullying. It is noteworthy that, because of the lack of previous studies exploring the first research question (i.e., are there differences in bullying representations between drawing and interview data?), we considered this aim of the study as eminently explorative. In regard to our second research question (i.e., are gender, and age, and bullying experiences related to children’s representations of bullying?), tentative hypotheses may be derived from previous literature based on interview and drawing data. More specifically, we hypothesized that gender differences might emerge as regards the type of bullying described by children (Naylor et al., 2006: Slee and Skrzypiec, 2015), with a prevalence of references to physical bullying being more frequent among males, and references to verbal bullying appearing more often among females (e.g., Naylor et al., 2006; Andreou and Bonoti, 2010); we also expected victims of bullying to be more likely to refer to direct forms of bullying (i.e., physical and verbal aggression) when compared to students who had not been involved in bullying (Naylor et al., 2006). Following studies based on self-report data we expected that age might also show some associations with the type of bullying mentioned, with a decline in the mentioning of physical bullying and an increase in references to verbal bullying with increasing age (e.g., Marengo et al., 2019).

## Materials and methods

### Sample

We recruited a convenience sample of 640 primary school students attending grade 4 to 5 in 7 different public primary schools located in North-West Italy. The mean age was 9.44 years (SD = 0.61; range = 8–12) and 53.3% of recruited students were male. All recruited students were fluent in Italian language, and none of the children presented diagnoses related to intellectual deficits or forms of psychopathology that would compromise their ability to participate in the research.

### Procedure

The aims of the research were presented in the classroom to the students by the one of the researchers. Participation in the research was on a voluntary basis and no reward was provided to the children, their families or the school. Participating children were administered a protocol that included, in order, the production of a drawing relating to their experience of bullying, a semi-structured interview and a questionnaire relating to their experience of involvement in bullying as a victim, aggressor or bully-victim. Typically, all assessments (i.e., drawing, interview, and self-report questionnaire) were performed on the same day for all students of a specific classroom. Before test administration, the children had the opportunity to familiarize themselves with the researchers. The researchers who administered the protocol were psychologists with experience in the field of developmental and school psychology, who had received training in child drawing administration and interpretation, and had extensive research experience.

### Ethical considerations

The study was approved by the IRB of the University of Turin (protocol no. 291061), and was undertaken in accordance with the indications of the Italian Association of Psychology (AIP) and the Helsinki Convention. After obtaining approval from the school headmaster, informed consent for participation in the research was obtained from both parents and children. The absence of informed consent from parents and children precluded the latter from participating in the research.

### Instruments

#### Children’s drawings

We involved students in a bullying drawing task in which children were asked to draw a picture portraying what bullying meant to them using the following standardized stem: “Please draw what bullying is like for you.” The children were given a white A4 sheet of paper and 12 colored crayons. No time limit was given to the children; however, children typically completed the task in less than an hour. Children completed the task in the classroom along with their peers; however, school desks were separated to avoid mutual interference. In contrast to other research (Bosacki et al., 2006), we did not ask the child to refer to his or her own experiences with bullying, but to describe what bullying was like for him or her by drawing it. In our opinion, this approach was instrumental in allowing the children to draw a more spontaneous representation of what he or she understood bullying to be.

#### Interview

Following the protocols used by Guerin and Hennessy (2002) and Bosacki et al. (2006), the authors developed a semi-structured interview designed to capture the children’s definition of bullying. During the interview, the authors asked the children what bullying means to them, what actions define bullying (i.e., types of bullying), what behaviors the victims engage in and what they feel emotionally when they are attacked, and whether other people (including children and teachers) are usually present when bullying takes place. Interviews were manually transcribed for later analyses.

#### Adolescent peer relations instrument

Children’s involvement in bullying and victimization was measured by administering an Italian adaptation of the adolescent peer relations instrument (APRI; Marsh et al. 2011) for the Italian context. The APRI is a psychometrically validated instrument that can be used to assess involvement in bullying behaviors as bullies and victims among school-aged children; although initially designed for use in adolescent samples, the APRI has also shown adequate functioning among children attending primary school (Finger et al., 2008). The Italian version of the APRI has shown good psychometric properties, as well as theoretically coherent associations with external criteria, including age, gender, internalizing and externalizing symptoms, and student-relationship quality, and students’ social status in the classroom (Marengo et al., 2019, 2021). The APRI consists of two sections, namely the bully and victimization sections, that can be combined to assess students’ involvement in bullying behaviors as a bully, victim, or bully-victim. The bully section consists of 18 items allowing for the scoring of three subscales representing three types of bullying, namely verbal (example item: “I made rude comments to a student”), social (example item: “I got my friends to turn against a student”), and physical (example item: “I hit or kicked a student hard”) bullying. Similarly, the APRI victim section consists of 18 items allowing for the scoring of three subscales respectively representing verbal (example item; “I was called names I didn’t like”), social (example item: “A student ignored me when they were with their friends”), and physical (example item: “I was hit or kicked hard”) victimization. Items are rated using a six-point Likert scale (1 = Never, 2 = Sometimes, 3 = Once or twice a month, 4 = Once a week, 5 = Several times a week, 6 = Daily). Based on responses to each subscale we generated three categorical variables grouping participants based on their involvement in each form of bulling/victimization, that is a distinction was made between uninvolved students and those involved as bully, victim, or bully/victims in each form of bullying (i.e., verbal, physical, and social bullying). For each type of bullying, in order to be identified as either bullies or victims, students needed to have indicated “Sometimes” or a higher frequency of involvement to at least one of the bullying or victimization items. Students were categorized as bully/victims if they responded “Sometimes” or a higher frequency of involvement to at least one item assessing bullying behaviors, and one item assessing victimization. Finally, uninvolved students were identified among those responding “Never” to all administered items.

### Data analysis

#### Content analysis of interview and drawing data

In order to detect relevant characteristics of bullying in collected data, a content analysis was conducted to develop a coding framework for subsequent analysis of the interview and drawing data. Please note that in looking into the data for such characteristics we followed a hybrid approach: first, based on a review of the literature, we determined a set of areas of interest, which we identified as being the following: (1) the specific type of bullying represented in the data; (2) the depiction/mentioning of specific criteria for bullying (i.e., repetition, power imbalance, and intentionality); (3) the behaviors and emotions shown by the victims; and finally, (4) the presence and behavior of other individuals on the scene. As a second step, for each of these areas of interest, we followed an inductive approach to let the characteristics emerge from the data. More specifically, the interview and drawing data were reviewed by one researcher in order to identify those characteristics reflecting differences in the type of bullying event described, the reference to theoretical criteria for bullying, the reactions and emotions of the victims involved, and the presence of other individuals in the scene.

In line with past qualitative studies examining children’s drawings of bullying (e.g., Bosacki et al., 2006), the bullying drawings were inspected for evidence of the presence of characters (graphical representations of one or more bullies or victims, as well as other people, including other children, and the teacher); size differences between the bully and the victim, single vs. multiple bullying scenes, the graphical depiction of verbal aggression (e.g., voice or speech bubbles and thought bubbles), physical bullying (e.g., kicking and punching); and of social/relational bullying (e.g., the exclusion/isolation of the victim, and the spreading of rumors against the victim, as depicted through voice or speech bubbles). Compliance with theoretical criteria for bullying (i.e., repetition, power imbalance, and intentionality) was determined based on combinations of the aforementioned characteristics (for example, a difference in size between the bully and the victim was considered indicative of a power imbalance; the presence of multiple bullying scenes representing the same characters was considered indicative of repetition over time; word bubbles).

A similar approach was employed in examining interview data. However, instead of looking for graphical representations of the aforementioned characteristics, we searched the interview transcripts for verbal references indicating the presence of characters, theoretical bullying criteria (i.e., repetition, power imbalance, and intentionality), the victim’s emotions and behavior in responding to the aggression by the bully, the type of aggression, and the involvement of other people beyond the bully and the victim during the bullying event.

**FIGURE 1:**
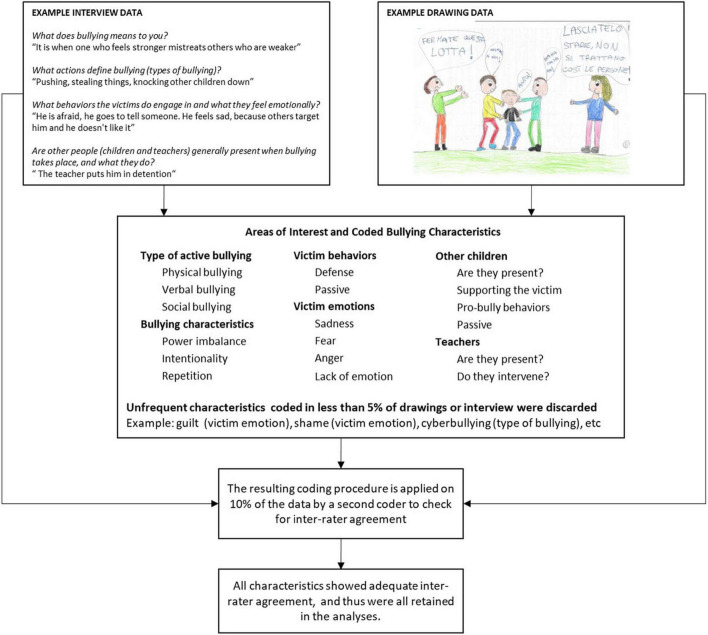
Diagram representing the coding of bullying characteristics in drawing and interview data.

The detected characteristics were then adapted for use as a coding framework. The reliability of the classifications was tested by checking the correspondence between the coded characteristics and an additional independent coding performed by a second researcher using the same coding framework. Independent coding was performed on a random sample representing 10% of the original interview and drawing data set. The percent agreement between two independent coders was calculated, with 70% agreement considered the minimum acceptable level of agreement. Results showed moderate-to-high agreement between coders, with the coding of sadness in the drawings showing the lowest agreement (78.6% agreement, Cohen’s *K* = 0.45) and coding of physical bullying in the interview data showing the strongest agreement (82.5% agreement, Cohen’s *K* = 0.71). Of the identified characteristics, only those that occurred in at least 5% of the sample were selected for further analysis (see Table 1). Figure 1 provides a diagram including example interview and drawing data, as well as a schematization of the procedure used to code the bullying characteristics.

**TABLE 1:** Difference in the distribution of coded characteristics in interview and drawing data.

Coded characteristics	Interview	Drawing	McNemar’s test	
	%	%	χ^2^	*p*
**Type of active bullying**				
Physical bullying	86.41	52.19	180.70	<0.001
Verbal bullying	86.72	55.31	164.61	<0.001
Social bullying	61.41	42.03	53.65	<0.001
**Bullying criteria**				
Power imbalance	25.00	26.25	0.23	0.634
Intentionality	8.13	5.78	2.48	0.115
Repetition	12.81	2.34	50.07	<0.001
**Victim behaviors**				
Defense	49.53	27.34	68.09	<0.001
Passive	63.28	64.53	0.20	0.658
**Victim emotions**				
Sadness	48.13	24.06	80.72	<0.001
Fear	15.63	10.63	7.75	0.005
Anger	8.59	1.41	31.64	<0.001
Lack of emotion	0.94	19.22	109.40	<0.001
**Other children**				
Are they present?	86.41	37.81	259.03	<0.001
Supporting the victim	61.25	6.88	327.20	<0.001
Pro-bully behaviors	15.78	28.75	33.79	<0.001
Passive	33.59	5.16	153.09	<0.001
**Teachers**				
Are they present?	65.62	4.69	369.08	<0.001
Do they intervene?	63.44	3.44	370.43	<0.001

*Continuity correction applied.

#### Quantitative analysis

First, we examined possible differences between interview data and the drawing in the prevalence of the emerging bullying characteristics. More specifically, we used McNemar’s test for paired nominal data to detect differences in the frequency of coded characteristics depending on the type of data collection, i.e., face-to-face interview and drawing.

Next, we examined associations between bullying characteristics and gender, age, and self-reported involvement in physical, verbal, and social bullying. Associations between bullying characteristics and gender and age were examined using the Pearson correlation coefficient. The association between self-reported involvement in bullying and the bullying characteristics was examined using logistic regression. More specifically, for each form of bullying (i.e., physical, verbal, and social), a categorical variable representing different roles students may take in bullying, namely, uninvolved, victim, bully, and bully/victim, was included in the model as a categorical factor, with the uninvolved group serving as the reference. In the tested models, the bullying characteristics were examined as dichotomous dependent variables, coded as follows: Characteristic is present in the data = 1, Characteristic is not present = 0.

## Results

### Differences in the distribution of bullying characteristics in the interview and drawing data

Table 1 shows the frequency distribution of coded characteristics in the interview and drawing data, as well as the results of McNemar’s test for differences in the distribution of characteristics between the two data collection methods.

Overall, all forms of bullying, i.e., physical, verbal, and social bullying, were more likely to be detected in the interview data than in the drawing data. Interestingly, social bullying was the least likely to be detected in both survey methods.

In terms of theoretical criteria for bullying (i.e., repetition, power imbalance, and intentionality), the presence of a power imbalance between the bully and victim was most frequently detected in both the interview data and the drawing data. In addition, we did not find differences in the prevalence of power imbalance and intentionality of bullying behavior between interview and drawing data, while the repetition criterion was found with higher prevalence in the interview data compared to the drawing data. It is worth noting that mentions of these bullying criteria had low prevalence in the data, as they were only found in the data of a minority of students, regardless of the method used.

In terms of behavior, victims were more often described as passively responding to bullying than exhibiting defensive behavior in both the interviews and the drawings. We note, however, that victims were more often described as defensive in the interview data than in the drawing data. There were no differences between interview and drawing data in the representation of the victim as passive.

Regarding the emotions shown by victims, the interview data shows that sadness was the most frequently reported emotion, followed by fear, anger, and a lack of emotions. In the drawing data, sadness also showed the highest prevalence, followed by a lack of emotion, fear, and anger. There were significant differences in the frequency of emotions across data collection methods: victims were more likely to be associated with sadness, fear, and anger in the interview data than in the drawing data; in turn, victims were more likely to be associated with a lack of emotions in the drawing data than in the interview data.

Students mentioned the presence of other children more frequently in the interview data than in the drawing data. Other children were more likely to be supportive or passive of the victim in the interview data than in the drawing data. In turn, children were more likely to exhibit pro-bullying behavior in the drawing data compared to the interview data. Regarding the role of teachers, they were more likely to be reported as present and intervening to support the victim in the interview data when compared to the drawing data.

### Associations between age, gender, and bullying characteristics in interview data

Table 2 shows the correlation between the bullying characteristics as detected in the interview data and both age and gender of the students. The significant correlations that emerged were all either small or negligible (*r* < 0.10). First, we found that the representation of physical bullying had a low negative correlation with female gender, corresponding to a low positive correlation with male gender. Verbal bullying and the presence of other children supporting the bully with bullying-promoting behaviors were positively correlated with both female gender and age. Age also showed a positive correlation with the representation of bullying as involving power imbalance between the bully and the victim, and a negative correlation with the victim’s expression of anger, although both of these correlations were negligible. Finally, age showed a small negative correlation with the mere presence of teachers when bullying took place, as well with the presence of teachers showing supportive behaviors toward the victim during bullying events.

**TABLE 2:** Interview data: correlation between bullying characteristics, age, and gender.

Interview	Age	Gender (female)

**Coded characteristics**	***r***	***r***
**Type of active bullying**		
Physical bullying	−0.04	−0.11**
Verbal bullying	0.13**	0.15**
Social bullying	0.03	0.04
**Bullying criteria**		
Power imbalance	0.09*	0.00
Intentionality	0.03	.00
Repetition	0.04	0.05
**Victim behaviors**		
Defense	−0.05	−0.05
Passive	0.03	0.05
**Victim emotion**s		
Sadness	−0.06	0.05
Fear	−0.06	−0.01
Anger	−0.08*	−0.01
Lack of emotion	0.03	0.08
**Other children**		
Are they present?	0.02	0.02
Supporting the victim	−0.01	−0.04
Pro-bully behaviors	0.12**	0.10**
Passive	0.06	0.06
**Teachers**		
Are they present?	−0.10**	−0.04
Do they intervene?	−0.11**	−0.05

*p < 0.05, **p < 0.01.

### Associations between age, gender, and bullying characteristics in drawing data

Table 3 shows the correlation between the bullying characteristics detected in the drawing data and the age and gender of the students. Significant correlations emerged, but these were either small or negligible in size (*r* < 0.10). First, we found that physical bullying had a small negative correlation with female gender, which corresponded to a small positive correlation with male gender. Verbal bullying, on the other hand, was positively correlated with female gender and age. Female gender also showed a positive correlation with students’ drawing of social bullying and victims expressing sadness. Female gender, in turn, was negatively correlated with the depiction of victims being attacked by a bully, which corresponded to a low positive correlation with male gender.

**TABLE 3:** Drawing data: correlation between bullying characteristics, age, and gender.

Drawing	Age	Gender (female)

**Coded characteristics**	***r***	***r***
**Type of active bullying**		
Physical bullying	−0.09*	−0.20**
Verbal bullying	0.12**	0.25**
Social bullying	0.06	0.11**
**Bullying criteria**		
Power imbalance	0.04	−0.05
Intentionality	−0.02	0.02
Repetition	0.06	0.04
**Victim behaviors**		
Defense	−0.05	−0.04
Passive	0.02	−0.005
**Victim emotions**		
Sadness	0.07	0.24**
Fear	−0.04	−0.06
Anger	0.02	−0.01
Lack of emotion	0.05	0.02
**Other children**		
Are they present?	−0.01	0.07
Supporting the victim	−0.01	0.07
Pro-bully behaviors	−0.01	0.05
Passive	0.02	0.00
**Teachers**		
Are they present?	−0.00	−0.06
Do they intervene?	−0.01	−0.08

*p < 0.05, **p < 0.01.

### Associations between physical, verbal, and social bullying roles and bullying characteristics coded in interview and drawing data

Tables 4–6 show the results of the logistic regression analyzes examining the associations between self-reported physical, verbal, and social bullying roles and the characteristics coded in the interview and drawing data. Only the results of models showing significant effects, grouped by the specific form of bullying, are reported in the tables. In all models, uninvolved students served as the reference group for estimating the effects of student involvement in bullying as victim, bully, and bully/victim.

**TABLE 4:** Logistic regression: regression coefficients and odds ratio for physical bullying roles predicting coded characteristics in interview and drawing data.

	Interview data				Drawing data			
	Physical bullying roles^a^				Physical bullying roles^a^			
Bullying characteristics	Victim	Bully	Bully/victim	*R* ^2b^	Victim	Bully	Bully/victim	*R* ^2b^
**Type of active bullying**								
Physical bullying	–	–	–	–	0.16 (1.17)	0.30 (1.350)	0.45* (1.57)	0.01
Verbal bullying	–	–	–	–	−0.30 (0.74)	−0.16 (0.852)	−0.52* (0.5)	0.01
**Bullying criteria**								
Power imbalance	–	–	–	–	0.46 (1.58)	0.56 (1.751)	0.58* (1.79)	0.02
Repetition	–	–	–	–	2.00 (7.39)	2.65* (14.154)	1.91 (6.75)	0.06
**Victim behaviors**								
Passive	−0.31 (0.73)	1.31* (3.72)	−0.25 (0.77)	0.03	–	–	–	–
**Victim emotions**								
Sadness	–	–	–	–	−0.36 (0.70)	−0.29 (0.748)	−0.60* (0.55)	0.02
**Other children**								
Supporting the victim	0.44* (1.55)	−0.08 (0.92)	0.23 (1.26)	0.01	–	–	–	–
Pro-bully behaviors	–	–	–	–	0.38 (1.46)	1.10* (3.004)	0.23 (1.26)	0.020
**Teachers**								
Are they present?	–	–	–	–	1.62* (5.05)	0.82 (2.27)	1.69* (5.42)	0.054

Values reported in parentheses are odds ratio.

*p < 0.05.

^a^Reference group is uninvolved students.

^b^Nagelkerke pseudo R^2^ is reported.

**TABLE 5:** Logistic regression: regression coefficients and odds ratio for verbal bullying roles predicting coded characteristics in interview and drawing data.

	Interview data				Drawing data			
	Verbal bullying roles^a^				Verbal bullying roles^a^			
Coded characteristics	Victim	Bully	Bully/victim	*R* ^2b^	Victim	Bully	Bully/victim	*R* ^2*b*^
**Type of active bullying**								
Physical bullying	–	–	–	–	0.13 (1.14)	0.12 (1.13)	0.45* (1.56)	0.01
Social bullying	0.29 (1.34)	0.66* (1.94)	0.53* (1.71)	0.02	–	–	–	–
**Bullying criteria**								
Intentionality	–	–	–	–	0.43 (1.54)	0.07 (1.08)	0.50* (1.64)	0.01
**Victim behaviors**								
Passive	−0.38 (0.69)	0.77* (2.15)	−0.03 (0.97)	0.03	–	–	–	–
**Victim emotions**								
Fear	–	–	–	–	−0.30 (0.74)	−0.09 (0.91)	−0.63* (0.53)	0.02

Values reported in parentheses are odds ratio.

*p < 0.05.

^a^Reference group is uninvolved students.

^b^Nagelkerke pseudo R^2^ is reported.

**TABLE 6:** Logistic regression: regression coefficients and odds ratio for verbal bullying roles predicting coded characteristics in interview and drawing data.

	Interview data				Drawing data			
	Social bullying roles^a^				Social bullying roles^a^			
Coded characteristics	Victim	Bully	Bully/victim	*R* ^2 b^	Victim	Bully	Bully/victim	*R* ^2 b^
Type of active bullying								
Physical bullying	–	–	–	–	0.19 (1.21)	0.47 (1.61)	0.54* (1.71)	0.01
Bullying criteria								
Power imbalance	0.79 (2.20)	1.12* (3.05)	0.35 (1.42)	0.02	–	–	–	
Victim emotions								
Fear	–	–	–	–	−0.01 (0.99)	0.21 (1.23)	0.81* (2.25)	0.03
Other children								
Supporting the victim	–	–	–	–	−0.70 (0.49)	−1.64* (0.19)	−0.49 (0.61)	0.03
Pro-bully behaviors	–	–	–	–	0.32 (1.39)	0.91* (2.484)	0.51 (1.66)	0.02

Values reported in parentheses are odds ratio.

*p < 0.05.

^a^Reference group is uninvolved students.

^b^Nagelkerke pseudo R^2^ is reported.

Regarding the association between characteristics coded in the interview and drawing data and self-reported physical bullying roles, Table 4 shows some significant effects. Students who reported being involved in physical bullying were more likely than uninvolved students to describe victims as passive in interview data. Students who reported being victims of physical bullying when responding to the questionnaire were more likely than uninvolved students to report the presence of other children supporting the victim in interview data.

Regarding the effects emerging from the analysis of characteristics detected in the drawing data, we saw that students who self-reported being victims of physical bullying were more likely to depict scenes of physical bullying and less likely to depict verbal bullying in their drawings compared to uninvolved students. Students who were involved in physical bullying as bully/victim were more likely than uninvolved students to draw bullying scenes depicting a power imbalance between bullies and victims. Students who described themselves as bullies (but not victims) were again more likely than uninvolved students to draw scenes depicting bullying events that were repeated in time, and in which other children exhibited pro-bully behaviors. Finally, students who were either victims or bullies were more likely than uninvolved students to draw teachers as present during bullying events.

Regarding the relationship between the characteristics emerging from interview and drawing data and self-reported verbal bullying roles, Table 5 shows some significant effects. Students who were classified as verbal bullies or bullies/victims were more likely to describe social bullying events in interview data than uninvolved students. Students who self-rated as verbal bullies were also more likely to describe victims as passive in interview data than uninvolved students. In terms of drawing data, students who self-reported being a verbal bully/victim were more likely than uninvolved students to draw scenes depicting physical bullying events and intentional bullying aggression, and less likely to show fear of victims.

Table 6 shows the relationship between characteristics coded in the interview and drawing data and self-reported involvement in social bullying roles. Students who self-reported being a social bully were more likely to describe a power imbalance between the bully and his or her victims during the interview than uninvolved students. In turn, students who self-reported being a bully/victim were more likely to draw scenes depicting physical bullying events and more likely to depict victims showing fear compared to uninvolved students. Finally, compared to uninvolved students, students who self-reported being social bullies (but not bully/victims) were more likely to draw children advocating for the bullying and less likely to draw children supporting the victim.

## Discussion

The first objective of this mixed-method study was to compare two approaches that can be used in qualitative research on bullying, i.e., interviews and the drawings, to determine if they are comparable as instruments to inform researchers and practitioners about children’s representation of bullying, or if they reflect different aspects related to this representation.

Based on our data, it appears that physical and verbal bullying is reported by children much more frequently than social bullying, both in the interviews and in the drawings. These data seem to be consistent with previous literature (Naylor et al., 2006; Andreou and Bonoti, 2010), which seems to indicate that physical and verbal bullying are the forms of peer victimization most frequently mentioned by children, while social exclusion is much less frequently told or reflected in drawings. However, comparing the two methods of data collection, it seems that the interview is more able to identify the different forms of bullying. This could be due to the fact that the child is able to provide more details and comments about bullying through the narrative.

In terms of the characteristics of bullying, our data show that the interview seems to be better at identifying the characteristics of repetition than the drawing. This could be due to the fact that repetition of bullying may be easier to express verbally than through a drawing. In general, however, we must point out that the criteria of power imbalance, repetition, and intentionality are poorly captured in both the drawing and the interview in our sample. This finding seems to be consistent with previous literature (Madsen, 1996; Guerin and Hennessy, 2002; Monks and Smith, 2006; Naylor et al., 2006) that informs us that these characteristics are not salient in children’s definitions of bullying, but rather shape adults’ definitions, starting with the researchers. In addition, the children tended to report more defensive victim behavior in the interviews than in the drawings. This could be due to the fact that they find it easier to describe these defensive behaviors in the interview than to depict them in a drawing.

The data also show that the victim’s emotions are central to the child’s representation of bullying. In particular, children tend to describe the victim as showing negative emotions, albeit significant differences in terms of the method of data collection emerged. While there is clear sadness in the interviews, the drawings tend to show a certain lack of emotion with increased frequency. This finding is curious and certainly deserves further investigation. However, the question of negative emotions related to the victim is consistent with the current literature, which has shown that the negative impact on the victim’s emotional well-being is a key element in defining bullying by children (Madsen, 1996; Naylor et al., 2006) and that they are more likely to report negative affect related to the victim in drawings and interviews (Bosacki et al., 2006; Naylor et al., 2006; Slee and Skrzypiec, 2015).

In the interviews, children tended to mention more often the presence of others besides the victim-bullies dyad, mentioning the presence of other children and teachers. In the drawings, on the other hand, the presence of other children is depicted much less frequently. Compared to the drawing, when responding to the interview children are more likely to describe teachers as people who intervene in the scene and other children as characters who intervene in favor of the victim or take a passive position towards the bully. In contrast, when drawing children tend to portray others as people who are likely to adopt a pro-bullying behavior. Still, it should be noted that the depiction of others in the drawing (teachers and children) is not common in our sample. This result seems to be consistent with the literature suggesting that children tend to focus more on the victim-bully dyad in drawings about bullying, and older children tend report more characters in the bullying scene (Bosacki et al., 2006). Overall, then, interview data appear to be a more informative qualitative survey tool than drawings when there is an interest in studying the representation of bullying among children. However, it seems that drawings can complement some information that is less clearly elaborated in interviews (e.g., negative emotions of the victim and behaviors conducive to bullying). Thus, this aspect seems to indicate that although interviews are to be preferred in the study of children’s representation of bullying, drawings can complement some relevant aspects and, therefore, it might be useful to combine both these qualitative techniques to get a more complete view of children’s representation of bullying.

In general, drawings have some advantages over interviews, as claimed by several authors (Butler et al., 1995; DiCarlo et al., 2000; MacPhail and Kinchin, 2004). Drawing is an attractive and enjoyable experience for children which allows them to express the elements of the drawing that are most important to them, and finally, it allows those children who are unwilling, unable, or too excited to express themselves verbally to express their views (Kukkonen and Chang-Kredl, 2018). Of course, the use of drawings also has limitations, particularly the fact that the quality of the representation is related to children’s artistic-graphic abilities and that they can only represent values that can be expressed visually (MacPhail and Kinchin, 2004). In this way, interviews can facilitate the description of bullying, as our data show, by overcoming the limitations of drawing. However, the interview could benefit from the addition of drawing to provide a more complete picture of the phenomenon.

The second objective of our study aimed at understanding if the children’s representations of bullying observed in the drawing and interview may be related to demographic characteristics of the children, as well as their direct involvement in bullying episodes as measured using a self-report questionnaire. Regarding gender, female gender seems to be positively associated with verbal victimization and negatively associated with physical victimization in both interviews and drawings. These data appear to be consistent with the literature that attributes greater involvement in physical bullying to males, while females are more likely to be involved in verbal bullying (Scheithauer et al., 2006; Kennedy, 2020). In addition, females are more involved in indirect forms of bullying than males (Mazzone et al., 2018). This finding is reflected in our analyses and is particularly highlighted by the interviews, which are therefore more informative regarding this form of victimization.

Females also tend to report more negative feelings of the victim, especially sadness, in drawings but not in interviews. This could be explained by a greater tendency of females to capture the victim’s emotional experience in the definition of bullying (Naylor et al., 2006; Byrne et al., 2016), but also by a tendency of females to portray more positive feelings in the drawings (Picard and Boulhais, 2011; Bozzato et al., 2021). However, future studies could clarify why this aspect was not captured in the interviews. Instead, the interviews in our survey show that females are more likely to report pro-bullying behavior in their definition of bullying. This finding is interesting and could be partly due to the fact that the female gender tends to maintain more harmony and closeness in social relationships (Rabaglietti et al., 2012; Sedgewick et al., 2019; Antonopoulou et al., 2021) and tends to exhibit more prosocial behavior (Van der Graaff et al., 2018). In this sense, bullying could be understood as an act that undermines attachment to others and can be seen as the opposite of prosocial behavior, which attracts greater attention in the female gender, who tend to recognize pro-social behavior more easily than males.

Some interesting correlations with age were found in the two methodological approaches. In both the drawings and the interviews, the depiction of verbal bullying increased with age, while only in the drawings a negative correlation emerged between physical bullying and age. Overall, these associations appear to be consistent with the developmental trajectory of peer victimization, whereby physically aggressive behaviors decrease with age in favor of verbal or indirect aggressive behaviors, likely as a result of the development of more sophisticated language and relationship tools (Longobard et al., 2019).

Finally, regarding age, the data collected in the interviews show us that older children tend to report less often the presence of the teacher in the bullying scene and to show more pro-bullying behaviors. In this sense, we must imagine that the peer group becomes the main social reference point for children as they get older and the place where they try to manage conflict situations among themselves in an increasingly autonomous way (Badenes-Ribera et al., 2019). This may explain the tendency to turn less to the teacher as they get older. As previous research (Bosacki et al., 2006) suggests, representations of bullying become more complex as children get older, which could likely explain a greater frequency of reference to bullying behaviors among older children.

Finally, we looked at possible associations between children’s participation in bullying as a victim, bully, or bully-victim and their representations of bullying in the form of drawings or interviews. There is virtually no data on this aspect in the literature, so there is a lack of references with which to compare our data. However, some significant associations between involvement in bullying and the characteristics of drawings and interviews emerged from the analyses. Regarding bullies-victims, the most significant data were found in the area of physical bullying. Children who self-reported being physically bullied were more likely to report the presence of other children supporting the victim in the interviews, while they reported the presence of the teacher in the drawings more than those who were not involved in bullying. Clearly, further study is needed to fully understand the significance of these data and why the same result is not evident in other forms of bullying victimization. However, we can hypothesize that physical violence is most prevalent among primary school children and may cause the most apprehension and anxiety due to the effects of aggression. In this sense, the presence of children supporting the victim or the presence of the teacher could be significant for children with previous experiences of physical victimization, as they could reduce the harm and stop the bully’s attack.

Some significant correlations also emerged for bullies. Those who reported physical or verbal bullying tended to describe the victim as passive during the attack more often than those who had not been bullies, while those who had committed social bullying tended to describe a greater power imbalance in interviews. Physical and verbal bullying are expressions of direct aggression. Overall, the data seem to reflect the tendency of the bully to select his victims by choosing them from among those who are weaker, less popular, and whose ability to defend themselves is seen as limited. In this sense, the interviews partially reflect the bullying experience from the perspective of the bully. Similarly, perpetrators of social bullying emphasize bullying-supportive behaviors to a greater extent, while they are less likely to identify support for the victim.

Bully/victim students differ from the other groups in that they self-report being both perpetrators and victims of bullying. Regardless of the form of bullying they have experienced, bully/victims were the group that more frequently depicted physical violence in their drawing. Bully/victim students tend to be described as dysregulated and are much more likely than others to engage in physically aggressive behavior and exhibit reactive aggression (Unnever, 2005; Yang and Salmivalli, 2013; Chung and Lee, 2020). It is possible, therefore, that the bullied child is more victimized in the school context, especially physically, and that this may influence his or her personal experiences with bullying, and ultimately the representation of bullying depicted in his/her drawing.

Bully/victim students tended to depict more frequently power imbalance (in the case of physical bullying) and intentionality (in the case of verbal bullying) in their drawings compared to individuals who were not involved in bullying. In addition, bully/victim students tended to express less fear in their drawings, especially in the case of physical and verbal bullying. Bully/victims tend to report more behavioral disturbances and aggressive behavior than bullies or victims or individuals not involved in bullying (Unnever, 2005), and the literature seems to indicate that these individuals tend to report less empathy (Zych et al., 2019) and fewer social and emotional skills (Habashy Hussein, 2013; Zych et al., 2019). Thus, it is likely that children who are victims of bullying, with less empathy and poorer social skills, tend to reflect victims’ emotional expressions less well. Different results were found for social bullying. Here, bully/victim students tended to represent fearful emotions of the victim more than non-bullies. This result is curious, and further studies will help to understand the differences. However, we could speculate that social bullying is an indirect form of bullying that does not require direct interaction with the victim, unlike physical and verbal bullying. In this sense, it is possible that perpetrators who engage in indirect bullying maintain better empathic skills, at least at the cognitive level, than perpetrators who engage in direct aggression (Yeo et al., 2011; Li et al., 2015). Further research is needed on this topic.

Finally, bully/victim students were more likely to report the presence of the teacher in their drawings compared to those who were not involved in bullying. However, they did not differ with respect to whether or not the teacher supports the victim when bullying takes place. This aspect is interesting because it seems to indicate some attention on the part of the bully/victims in relation to the presence of the teacher. We can imagine that this corresponds to the subjective experience of the bullying victim who attracts the attention of the teacher, living the dual role of victim and aggressor. In this sense, children who are victims of bullying tend to be poorly adjusted in school and receive negative attention from the teacher to an even greater extent than children who are bullies or victims (Haynie et al., 2001; Olweus, 2001; Marengo et al., 2018).

## Conclusion and limitations

To our knowledge, this was the first study that attempted to compare two different data collection methods in the qualitative domain (drawing and interview) in detecting bullying characteristics in the representation of bullying among primary school children. In summary, our data show that the interview appears to be more capable of detecting different forms of bullying and tends to be more informative about a variety of bullying-related characteristics. However, although the interview appears to be more informative in general, the two approaches also differ on the characteristics of elementary school children’s bullying representations. This is a new finding that suggests that it is useful to incorporate various qualitative techniques in the empirical study of bullying by children, especially since research on this topic seems to be dominated by the quantitative approach (Patton et al., 2017). In this direction, qualitative research can complement quantitative data and inform us about what aspects characterize children’s representations of bullying, which has a significant impact on prevention strategies and interventions. In addition, our study is the first attempt to examine an association between involvement in bullying as a victim, bully, or bully/victim and bullying-related characteristics captured by two qualitative instruments in the same survey. Overall, in addition to gender and age, our data found an association between experiences of bullying and several characteristics of the representation of bullying in drawings and in the interview. It is possible, therefore, that involvement in bullying, depending on the role and type of bullying behavior, may influence elementary school children’s representation of bullying. However, the literature on this point is very sparse, and further studies are needed to understand these relationships.

Clearly, the results of this exploratory study need to interpreted with caution, as it is important to consider the limitations of the study. Although we recruited a large sample, it cannot be claimed to be representative of the Italian child population. Future studies could therefore recruit representative samples and apply the same protocol to children and adolescents of different ages and cultures to increase the generalizability of the results and assess their cross-cultural consistency. In addition, we relied solely on child self-report to capture bullying involvement. Future studies could use third party informants, such as teachers or parents. Factors such as text comprehension and social desirability might have influenced subjects’ responses to the tests. Finally, the cross-sectional approach prevents us from expressing ourselves in terms of linear causality. Therefore, longitudinal studies will be able to clarify whether involvement in bullying affects the child’s representations as determined by the interview and drawing.

## Data availability statement

The raw data supporting the conclusions of this article will be made available by the authors, without undue reservation.

## Ethics statement

The studies involving human participants were reviewed and approved by the University of Turin. Written informed consent to participate in this study was provided by the participants or their legal guardian/next of kin.

## Author contributions

DM and MS: methodology, formal analysis, writing—original draft, and methods section. CL: conceptualization, writing—review and editing, and supervision. MF: conceptualization, collecting data, and writing—original draft. All authors contributed to the article and approved the submitted version.
